# Post-translational Modification Regulates Formation and Cargo-Loading of Extracellular Vesicles

**DOI:** 10.3389/fimmu.2020.00948

**Published:** 2020-05-22

**Authors:** Jonathan M. Carnino, Kareemah Ni, Yang Jin

**Affiliations:** Division of Pulmonary and Critical Care Medicine, Department of Medicine, Boston University, Boston, MA, United States

**Keywords:** PTM, EV, exosome, EV-cargo, EV-protein, EV-miRNA

## Abstract

Accumulating evidence suggests that post-translational modifications (PTMs) regulate the selective encapsulation of non-coding RNA molecules into extracellular vesicles (EVs) and contribute to the downstream functions of EVs or EV-cargo non-coding RNAs. EVs are a newly studied mechanism of intercellular communication that involves the transfer of molecules, including but not limited to proteins, lipids, and non-coding RNAs, to induce functional changes in the recipient cells. In this present mini-review, we focus on the PTM-regulated protein and non-coding RNA selection into eukaryotic EVs.

## Introduction

Cell-cell cross-talk is facilitated via multiple methods of intercellular communication, such as physical interactions via the formation of tunneling nanotubes and/or the secretion of soluble factors (1). In the past two decades, emerging evidence has shown that EVs serve as an important component of the intercellular exchange of information. EVs carry multiple signaling molecules including bioactive lipids, proteins, and nucleic acids. Besides transporting signaling molecules among cells, EVs are also responsible for the removal of harmful cellular components (2–4)). Accumulating evidence indicates that EV cargo is selectively, rather than randomly, encapsulated into the vesicles. In this mini-review, we focus on the role of PTMs in the regulation of encapsulating intracellular proteins and cellular nucleotides, particularly non-coding RNA molecules, into eukaryotic EVs.

## History and Nomenclature

Historically, the production of membrane-bound vesicles by cells has been well-known as a universally conserved process occurring in both prokaryotes and eukaryotes (5–8). EVs were originally discovered in the 1960s when researchers used high-speed centrifugation to isolate a precipitate from blood which was confirmed to reverse coagulation dysfunction in a manner similar to the fraction of thromboplastic protein (9). Between the 1970s and 1980s, multiple studies proposed that membrane-like vesicles existed in solid tissues, physiological fluids, and cell culture media (10, 11). Finally, in the 1990s, the discovery that B-cells infected by the Epstein-Barr virus were able to secrete molecules (EVs) for the enrichment of T lymphocytes in the immune system lead to the idea of EVs functioning in cell to cell communication (12, 13). From this point, studies into the function of EVs in many physiological and pathological processes became more common. In recent years, our understanding of extracellular vesicles and their biological importance has progressed, and the International Society of Extracellular Vesicles has continuously released updated guidelines throughout this time to guide researchers in this novel field.

**Figure 1:**
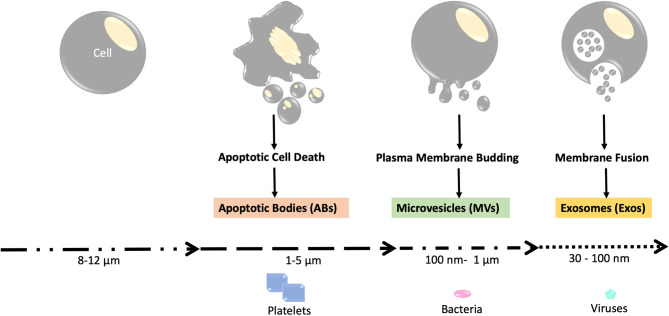
Schema of the three major categories of EVs. Apoptotic bodies, which are released during apoptotic cell death, are the largest form of EVs with sizes comparable to that of platelets (1–5 μm). Microvesicles are medium-sized EVs that are similar in size to bacteria (around 100 nm to 1 um) and are produced through plasma membrane budding. Exosomes are the smallest type of EVs with sizes ranging from 30 to 100 nm. The exosomes are first formed as intraluminal vesicles (ILVs) in membrane bound vesicles (MVBs). Production of ILV/MVB-mediated EV involves proteins such as the endosomal sorting complexes required for transport (ESCRT) and lipids. The MVBs are then fused with lysosomes or the plasma membrane for extracellular release, which involves RAB proteins (RAB11, RAB27, and RAB35).

Previously, EVs were categorized based on their sizes, mechanism of generation, and surface markers. Based on these criteria for classification, the three major categories for EVs were apoptotic bodies (ABs), microvesicles (MVs), or exosomes (Exos). The size of the largest EV, i.e., apoptotic bodies (AB) (14) is in the range of microns, almost similar to the size of mammalian platelets (Figure 1). Apoptotic bodies were regarded as the vesicles produced by dying cells undergoing apoptosis, and their size ranges between 50 to 5000 nm (15). Microvesicles, which form by the outward budding of the plasma membrane, ranged in size between 100 and 1000 nm (16). Lastly, Exosomes were named as the vesicles formed within the cell as multivesicular bodies and subsequently released by fusion with the plasma membrane; their size ranges from 30 to 150 nm (13). However, due to the significant size overlap among these categories of EVs, this nomenclature has been discontinued by International Society of Extracellular Vesicles (ISEV), despite frequently being used in current literature (Figure 1).

According to the current guidelines provided by ISEV, EVs can be named after physical features of EVs like size (small EVs or sEVs, medium/large EVs or m/lEVs), biochemical properties (CD63+/CD81±EVs, Annexin A5-stained EVs, etc), or by description of cellular condition and the origin cell (podocyte EVs, hypoxic EVs, large oncosomes, apoptotic bodies). Currently, EVs can be recovered by medium speed centrifugation (10,000 × g) or by ultracentrifugation (100,000 × g). EVs isolated using centrifugation at medium speed can be referred to as large oncosomes, ectosomes, microvesicles, or large/medium EVs. EVs isolated using ultracentrifugation are often named small EVs or exosomes (17, 18) (Figure 1).

## Mechanism of EV Generation

At least three distinct mechanisms have been proposed for EV biogenesis. ABs are often generated during the process of apoptotic cell death via membrane disintegration. Therefore, most studies are focused on the non-AB EVs, which are smaller than 1 μm. For the smaller EVs (mainly MVs and exosomes in the previous nomenclature), the pathway of biogenesis includes direct budding from the plasma membrane (often used by MVs) and the formation of intraluminal vesicles within multivesicular bodies (MVBs) (often used by exosomes). Endosomal sorting complexes required for transport (ESCRT) protein has been reported to be involved in intraluminal vesicle/MVB-mediated EV generation, while lipid raft proteins caveolin-1 (cav-1) have been shown to participate in MV generation via pinching off the plasma membrane (19–21) (Figure 1). Exosome and MV biogenesis may also share some elements in their molecular machinery.

Since EVs share the same plasma membrane with their parent cells, PTMs of the vesicle membrane proteins have often been identified. Currently, it is believed that the PTM of membrane proteins or proteins associated with the MVB-ER (multivesicular body-endoplasmic reticulum) system plays an important role in MV or Exosome formation, subsequently, promotes intercellular communications in the development of disease process.

## EV Cargo and Function

EVs are known to transport potential signaling molecules among cells and deliver these different signals to recipient cells which may modify their function and phenotype. The ability of EVs to transfer “messages” between cells means they could serve as potential biomarkers for diseases in the near future. Recent studies have uncovered that EVs play critical roles in the pathogenesis of various diseases such as asthma, COPD, allergies, idiopathic pulmonary fibrosis, and cancer (16). Currently, the most often studied signaling molecules are small non-coding RNAs and proteins. Accumulating data suggests that this EV-cargo is not selected and encapsulated into the EVs randomly. Instead, it is selected specifically based on the cell of origin, functional status of the “mother” cell, and noxious stimulation (22, 23). For example, macrophages in response to bacterial infection actively remove intracellular microRNA(miRNA)-223/142 via an EV-mediated manner. Subsequently, reduced levels of intracellular miR-223/142 lead to pro-inflammatory activation of macrophages (3). It is already well-recognized that EV-cargo is actively selected, despite this, the detailed mechanisms by which EV-cargo is selected remains incompletely understood. Of note, emerging evidence indicates that post-translational modifications (PTMs) are one of the key factors which mediate the selection of certain EV-cargo, such as proteins and RNA molecules.

## Common Type of Post-Translational Modifications (PTMs) of EV Protein

The chemical changes of proteins after translation are often catalyzed by enzymes which identify unique sequences in the target proteins. These chemical modifications are referred to as PTMs. PTMs include, but are not limited to cleavage of precursors, formation of disulfide bonds or covalent addition or removal of low-molecular-weight groups (24). The common low-molecular-weight groups are listed in Table 1. In the cells, protein degradation and *de novo* synthesis often take much more time and energy. Some PTMs are readily reversible and therefore allow for rapid modification of protein function. PTMs covalently add a functional group to a protein which influences the function and properties of proteins. PTMs engage in almost all cellular events, including the formation, function, and cargo composition of EVs.

**Table 1:** List of all post-translational modifications.

**Name of PTM**	**Chemical modification**
Acetylation	Attaching acetyl group (CH_3_CO)
Amidation	Attaching amide group (–NH2) C-terminal
Biotinylation	Covalently attaching biotin
Cysteinylation	Forming disulfide bonds between free Cys molecules
Deamidation	Removing or converting amide group
Farnesylation	Adding an isoprenyl group to a cysteine residue
Formylation	Addition of a formyl functional group
Geranylgeranylation	Adding 1 or 2 twenty carbon lipophilic geranylgeranyl isoprene to Cys
Glutathionylation	Adding glutathione to Cys
Glycation	Covalently attaching a sugar to a protein or lipid
Glycosylation	Enzymatically attaching glycans to proteins
Hydroxylation	Introducing a hydroxyl group (-OH) into an organic compound
Methylation	Adding a methyl group
Mono-ADP-ribosylation	Adding ADP-ribose to arginine side chains
Myristoylation	Covalently attaching myristoyl group to an N-terminal glycine residue
Oxidation	Substance is oxidized by giving away electrons
Palmitoylation	Covalent attaching fatty acids to cysteine, serine or threonine
Phosphorylation	Attaching a phosphoryl group
Poly(ADP-ribosyl)ation	Covalently attaching polymers of ADP-ribose to protein
Stearoylation	Covalently attaching stearic acid to a protein
Sulfation	Enzyme-catalyzed conjugation of a sulfo group

An EV-mediated rapid removal of selected intracellular molecules has been reported as another way of controlling function (3). Regulation of EV components into and out of EVs is essential for the effects they have on biological functions in recipient cells under physiological and pathophysiological conditions. Incorporating the selected molecules into EVs requires rapid and potentially reversible processes. Emerging evidence suggests that PTMs participate in the process of incorporating intracellular molecules into EVs, and subsequently their release from cells. We will review several well-reported PTMs below.

## Phosphorylation

Phosphorylation is one of the most commonly studied PTMs and has been demonstrated to regulate multiple cellular processes and signaling pathways. It plays a crucial role in protein function, folding, and subcellular localization (25). A large scale phosphoproteomic study by Gonzales et al. showed 19 phosphorylation sites corresponding to 14 phosphoproteins in human urinary exosomes (26). In cultured SW620 colon cancer cells, more than 300 phosphoproteins containing at least 1,000 phosphosites have been recognized in exosomes (27). In exosomes, high levels of tyrosine (Y)-phosphorylated sites in, specifically 6.4% in exosome and just 0.6% in the cell, are observed in SW620 cancer cell-derived exosomes, suggesting that the tyrosine phosphorylation of exosomal proteins may contribute to exosome formation and functions. The phosphorylated exosomal proteins may also serve as a biomarker for certain types of malignancy. In one large study of EV protein phosphorylation, EVs from samples of human plasma were found to contain nearly 10,000 phosphopeptides. Among these, over 9,000 phosphopeptides or 1,900 phosphoproteins are from MVs and ~1,000 phosphopeptides or more than 400 phosphoproteins are from exosomes, respectively (27). The authors further found that when comparing with normal controls, in plasma EVs, ~144 phosphoproteins are markedly higher in breast cancer patients. As reported by Chen et al., ~7,000 phosphopeptides are detected in 1 mL of plasma, further suggesting the potential roles of EV-phosphorylated proteins as a biomarker for human diseases (28).

Phosphorylation which is observed on individual proteins from EVs has been demonstrated to play a pivotal role in regulating the EV formation and EV-cargo selection. For example, cell surface protein caveolin-1 (cav-1), after phosphorylation at its tyrosine 14 (pY14), exposes its cav-1 scaffolding domain (CSD) to interact with the hnRNPA2B1. HnRNPA2B1 is a well-known RNA binding protein. The cav-1/hnRNPA2B1 complex subsequently is incorporated into the EVs, along with the hnRNPA2B1-bound miRNAs (29). Phosphorylation and O-GlcNAcylation of cellular proteins have been reported to mediate the incorporation of selected miRNA molecules into EVs. RNA-binding protein hnRNPA2B1 (hnRNPA2B1) is shown to selectively transport miR-17 and −93 into EVs (30). Lee et al. (29) further demonstrated that hnRNPA2B1 underwent O-GlcNAcylation. O-GlcNAcylation of the RNA-binding region at its serine 73 and serine 90 is in part responsible for the hnRNPA2B1–miRNA interactions. This is an example of how PTMs (phosphorylation and GlcNAcylation) regulate miRNA molecules being secreted by cells via EVs.

## Ubiquitylation

Ubiquitylation is a ubiquitous post-translational modification found in the majority of eukaryotic cells. During the process of ubiquitylation, ubiquitin, a small regulatory protein, is attached to the lysine residues on a target protein through an isopeptide bond. The E1, E2, and E3 ligase family is responsible for the reaction of ubiquitylation (31, 32). First, ubiquitin is activated by E1 enzymes, then conjugated by E2 enzymes, followed by attachment to the substrate protein by E3 enzymes (ubiquitin ligases) (33–35). After endosomal proteins are ubiquitylated and deposited into the lumens of multivesicular bodies (MVBs), they are removed by either lysosome-mediated degradation or secreted out of the cells as exosomes.

Ubiquitylation has been well-reported to participate in EV-cargo selection and EV-generation. For example, it has been reported that protein cargo packaged into urinary exosomes is ubiquitylated and can be recognizable by the ESCRT complex on MVBs. Additionally, 13% of the proteins found in exosomes are ubiquitylated, and 21% of the ubiquitylated proteins in exosomes are transmembrane proteins. Among all the ubiquitylated proteins in urinary exosomes, a large number of them are involved in transcription/transcriptional regulation (36). Ubiquitin is cleaved from the ubiquitinylated proteins during the process of cargo incorporation by certain deubiquitylases (37). In 1 mg of exosomal peptides isolated from human urine, >600 ubiquitylated proteins have been detected (37). This observation suggests that exosome formation does not require deubiquitylation (37).

Ubiquitylated proteins from cell lysates often show smeared bands when detected using Western Blot analysis. Interestingly, the ubiquitinated exosomal proteins only have discrete bands (38). Currently, it is unclear whether these exosomal proteins are dominantly mono-ubiquitinated or polyubiquitinated. It is also possible that these proteins are initially sorted as polyubiquitinated and some of the ubiquitinated proteins are deubiquitylated during the exosomal pathway. Alternatively, it's possible that selected monoubiquitinated protein is enriched in exosomes and the levels of other polyubiquitinated exosomal proteins are too low to be detected (38). In EVs, all of the machinery necessary for ubiquitination has been identified by proteomics, such as E1, E2, and E3 ligase family (39). Mono-ubiquitinated endocytosed proteins may be trafficked to EVs as a normal mechanism of protein recycling. Normally, secreted proteins may be repackaged for extracellular release. It is thought that during the process of endocytosis/phagocytosis, the purpose of ubiquitination is to tag a protein for delivery to EVs (7, 36). For example, deubiquitinated HIV Gag potentially is sorted into a lysosomal pathway for degradation, whereas ubiquitinated HIV Gag is potentially packaged into an exosome which has been hijacked by HIV for its budding. Currently, COP9 signalosome regulatory complex (COP9)-associated subunit 5 (CSN5) machinery has been reported to be responsible for driving exosomal protein sorting via ubiquitination and de-ubiquitination axis (38, 40).

Despite being well-reported in EV-cargo proteins, the ubiquitination is not required for all proteins to be incorporated into EVs. For example, ubiquitination of MHC-II or heat shock protein 70, is not a prerequisite for its incorporation into exosomes (38, 41). The ubiquitylated protein can be deubiquitylated on its route to EVs. It is believed that ubiquitylated proteins are recruited to multi-vesicular bodies (MVBs) and only a portion, without earlier deubiquitylation, are appropriated into intraluminal vesicles (ILVs) before incorporation into EVs. Currently, ubiquitin is thought of as a temporary ESCRT-interaction domain which may be added and removed from proteins in order to control their interaction with the ESCRT machinery (42).

## Sumoylation

Sumoylation is a PTM that is analogous to ubiquitylation in terms of the enzyme classes and reaction processes (43). The difference between sumoylation and ubiquitylation is that sumoylation refers to adding small ubiquitin-like modifiers (SUMO), rather than adding ubiquitin itself (43–45).

Sumoylation has been reported to function as a sorting element in the release of proteins within EVs. Like ubiquitin, SUMO proteins bind to target proteins as part of a PTM system. However, unlike ubiquitin which mainly leads to degradation, SUMO protein participates in a variety of cellular processes, including but not limited to nuclear transport, transcriptional regulation, apoptosis, and protein stability (46, 47). After the last two amino acids of the carboxy-terminus have been cleaved off, SUMO becomes active. Recently, SUMO-dependent sorting of GFP, APP, and α-Synuclein into EVs have been identified (42, 48). SMT3 suppressor of mif two 3 homolog 2 (SUMO-2) belongs to the SUMO family and interacts with phosphoinositols. Emerging data have demonstrated how SUMO-2 interacts with PI3P and PI(3, 4, 5)P3, and subsequently interacts with ESCRT machinery (48). ESCRT-0 binds with the PI(3)P via its HrsFYVE domains and subsequently is recruited to sites of intraluminal vesicle formation (48). As mentioned above, EVs be derived from late endosomes/multivesicular bodies or by direct budding of the plasma membrane. PI3P is enriched at the endosomal membrane and PI(3, 4, 5)P3 primarily locates at the plasma membrane. Both PI3P and PI(3, 4, 5)P3 interact with ESCRT machinery. The fusion of cytosolic protein TyA and PI(3, 4, 5)P3-binding domain of AKT protein kinase quickly targets the protein to extracellular vesicle budding sites (49). This suggests that, instead of being sorted into multivesicular endosome-derived ILVs, SUMO-2 may bind to the plasma membrane for consequent shedding into vesicles (49). Spatial selectivity of SUMO-2 to PI(3, 4, 5)P3 binding may be caused by differences in ratio of cholesterol to phospholipid, which is greater at the plasma membrane compared to endosomal membranes (49).

Ubiquitin-like protein 3 (UBL3) is thought to play a part in the sorting of greater than half of all EV-cargo proteins. UBL3 modification is critical in the sorting of UBL3 to MVBs and EVs. In one report, 29% of the 1,241 UBL3-interacting proteins were believed to be EV-cargo protein. This finding suggests that UBL3 could have a role in the majority of sorting of all EV-cargo proteins (50).

## Glycosylation

Glycosylation facilitates correct protein folding and the EV membrane consists of abundant glycoproteins (51). The most commonly studied type of glycosylation is N-glycosylation. In human urinary EVs, 126 N-glycopeptides related to 37 glycoproteins have been identified. In breast cancer patients, 1,453 unique glycopeptides corresponding to 556 glycoproteins in EVs have been detected (27). Again, the elevated glycoproteins in breast cancer patients could potentially serve as a novel candidate for liquid biopsy. Glycans also relate closely to the export and uptake of EVs like the phosphorylation. Despite that O-glycosylation may be crucial in regulating EV cargo selection (29) and potentially serve as biomarkers in cancer patients. Currently, O-glycan sample preparation techniques and MS-based characterization methods are not efficient which make it difficult to study O-glycan in depth (52).

The heavily glycosylated form of the extracellular matrix metalloproteinase inducer (EMMPRIN) is a marker for pro-invasive EVs. In breast cancer patients, EMMPRIN is identified at high levels in EVs (53, 54). Of note, EV deglycosylation inhibits EV-induced invasion (53, 54). In addition, a highly glycosylated EMMPRIN is required for EV-stimulated cancer cell invasion (53, 54).

Glycosylation may be particularly useful for the potential clinical transformation of EV-mediated therapeutics. Targeting EVs to a specific cell surface receptor is often necessary for the effective delivery of therapeutic molecules *in vivo* (55). Glycosylation of a protein motif protects it from degradation. This method allows for the expression of targeting peptides on the exosome surface and prevents degradation. Therefore, glycosylation can potentially be useful when engineering exosomal surface proteins to enhance EV uptake or reorganization by the targeted recipient cells (55).

Glycosylation has a function in the sorting and expression of alternative exosomal proteins. For example, sorting of cell surface protein EWI motif-containing protein 2 (EWI-2) into exosomes relies on the presence of complex *N*-linked glycans on this protein (56). Mutation of only one of the three *N*-linked glycosylation sites robustly reduced EWI-2 expression in EVs, and mutation of every site deregulated both cellular and EV-associated EWI-2 levels.

## Deimination

Protein deimination is a permanent/irreversible PTM produced by the peptidylarginine deiminase (PAD) family of enzymes. In target proteins, PAD converts arginine into citrulline, which subsequently leads to protein structural and functional changes (57). Interestingly, PADs have been reported to regulate the release of EVs. It has been reported that 42 crucial metabolic and immune proteins are post-translationally deiminated in EVs only. Deiminated proteins in EVs have linked to Kyoto Encyclopedia of Genes and Genomes (KEEG) pathways of HIF-1 signaling and glycolysis (58). Additionally, deiminated proteins reported in EVs are enriched for both gluconeogenesis and glycolysis KEGG pathways.

## Acetylation, Palmitoylation, Uridylation, Phosphorylation and GlcNAcylation

Scattered reports have shown that acetylation and palmitoylation affect specific protein and/or RNA molecule secretion via EVs. For example, one of the HSP 70 family proteins, GRP78, can be acetylated resulting in the increase in interaction with VPS34, a phosphatidylinositol-3 kinase that generates unique complexes involved in EV transport (59).

Palmitoylation is a reversible PTM and is understood to serve as an anchor for proteins on the membrane. It has been reported to regulate the sorting and release of Latent membrane protein 1 (LMP1) of the Epstein-Barr virus *via* EVs (60).

Small RNA compositions in the secreted MVs are different from that in their parent cells. In addition to protein PTMs, 3′ end nucleotide additions have also been reported to mediate the selection of miRNAs for packaging into EVs. For example, Zhang et al. (3) reported that 3′ end uridylation, but not adenylation, mediates the packaging of miR-223/142 into MVs (3).

ISGylation is the covalent addition of Interferon-stimulated gene 15 (ISG15) protein, by an isopeptide bond, to cytoplasmic and nuclear proteins. Protein ISGylation inhibits the replication of many viruses. ISGylation also promotes the lysosomal degradation of MVB proteins and serves as a novel ubiquitin-like modifier in the regulation of exosome production (42).

## Potential Impacts of EV protein PTMs on the Pathogenesis, Diagnostics, and Therapeutics of Human Diseases

It has been well-documented that EVs play an essential role in intercellular and interorgan communication during the development of many human diseases (16). EVs in circulation or body fluid potentially serve as novel biomarkers for a variety of human diseases (13). EV protein PTMs often directly influence EV formation and signal transmission. Most PTMs discussed above participate in the process of EV formation and EV-cargo loading. Ubiquitylation and sumoylation are likely to facilitate EV degradation too. Glycosylation of vesicle membrane proteins is often involved in not only EV formation but also EV uptake by recipient cells and signal transduction. All of these PTMs are often stimulation- and cell type-dependent. Different stimuli result in distinct PTMs on a specific protein. For example, oxidative stress induces cav-1 tyrosine 14 (Y14) phosphorylation and facilitates the loading of a specific miRNA repertoire into EVs (29). On the other hand, bacterial infection has a greater impact on miRNA 3' end uridylation. This process leads to loading another group of miRNAs into secreted EVs (3).

### Summary and Challenges

Currently, the challenge of studying EV protein PTMs are mainly technical difficulties. First, the protein amount is low in EVs, making it extremely difficult to quantify and detect PTMs of EV proteins. Additionally, the process of isolating EVs can sometimes alter the PTMs, for example, ultracentrifugation may dissociate some temporary bonds of PTMs. Future studies will need to evaluate the impact different EV isolation methods have on PTMs. Furthermore, since EVs share the same plasma membrane as their parent cell, the large number of lipids, or phospholipids, cholesterols, etc., may affect the detection of protein PTMs.

In summary, EV protein PTMs play a pivotal role in regulating the EV formation, function, and EV-cargo selection. SUMOylation, Phosphorylation, Ubiquitylation, Glycosylation, and other PTMs all have been reported in the formation of EVs, EV uptake and EV-cargo loading.

EV-cargo is selectively incorporated into EVs. Noxious stimuli-induced PTMs play an important role in regulating protein or miRNA components for cell secretion via EVs. Besides the above mentioned, more PTMs may be further reported to regulate EV-cargo selection in future studies. PTM of EV proteins may shed a light on novel biomarker development for human diseases and provide novel therapeutic strategies.

## Author Contributions

JC and YJ wrote and edited the initial manuscript. JC made the table and KN made the new figure/figure legend. The final manuscript was revised by JC, YJ, and KN.

## Conflict of Interest

The authors declare that the research was conducted in the absence of any commercial or financial relationships that could be construed as a potential conflict of interest.
